# Effects of Defoliation on Phenolic Concentrations, Antioxidant and Antibacterial Activity of Grape Skin Extracts of the Varieties Blaufränkisch and Merlot (*Vitis vinifera* L.)

**DOI:** 10.3390/molecules24132444

**Published:** 2019-07-03

**Authors:** Valentina Pavić, Toni Kujundžić, Marina Kopić, Vladimir Jukić, Ulrike Braun, Florian Schwander, Mato Drenjančević

**Affiliations:** 1 Department of Biology, Josip Juraj Strossmayer University of Osijek, Cara Hadrijana 8/A, 31000 Osijek, Croatia; 2 Faculty of Agrobiotechnical Sciences Osijek, Josip Juraj Strossmayer University of Osijek, Vladimira Preloga 1, 31000 Osijek, Croatia; 3 Julius Kühn-Institut, Federal Research Centre of Cultivated Plants, Institute for Grapevine Breeding Geilweilerhof, 76833 Siebeldingen, Germany

**Keywords:** defoliation, Blaufränkisch, Merlot, phenols, antioxidant activity, antibacterial activity

## Abstract

*Vitis vinifera* L. has been highlighted by its many positive effects on human health, including antibacterial, anti-inflammatory and antioxidant activity due to its rich phytochemical content. Removing six basal leaves has great potential to influence the production of phytochemicals. The purpose of this study was to determine the impact of different terms of defoliation on the antioxidant and antibacterial activity of grape skin extracts of the Blaufränkisch and Merlot varieties. The total phenolic concentration, total and individual anthocyanin concentration, antioxidant activity and antibacterial activity on gram-positive and gram-negative human pathogens have been determined. The study was conducted on three treatments (control treatment without defoliation, defoliation immediately after bloom and defoliation before the start of the *véraison* phase). The results showed statistically significant enhancement of the total phenolic concentration as well as the antioxidant and antibacterial activity in both studied cultivars. Defoliation just after blooming was the preferable defoliation term in the Merlot variety for achieving the highest total anthocyanin concentration, antioxidant activity and significant increase of antibacterial activity against all four investigated bacteria. Defoliation before the start of the *véraison* phase was the preferable defoliation term for achieving the highest total anthocyanin concentration in the Blaufränkisch variety. In general, treatment of defoliation immediately after bloom was more beneficial compared with the defoliation before the start of the *véraison* phase.

## 1. Introduction

Polyphenols are phytochemicals found extensively in fruits, vegetables and beverages. As secondary plant metabolites, they are commonly involved in defense against ultraviolet radiation or pathogen attack [1]. Epidemiological studies and combined meta-analyses had suggested that extended consummation of diets rich in plant polyphenols give certain protection against the occurrence of cancers, cardiovascular diseases, diabetes, insulin resistance, obesity, neurodegenerative diseases and osteoporosis [2,3]. The overriding class of biologically active compounds in grapes are polyphenols involving flavonoids, stilbenes and proanthocyanidins. Among the polyphenols found in grapes, flavonoids are the most plentiful biologically-active phytonutrients, possessing cardioprotective, neuroprotective, antimicrobial and antiaging properties [4,5,6,7]. Brightly colored grapes are especially rich in anthocyanins and they have been known for the inhibition of lipid peroxidation and cyclo-oxygenase (COX)-1 and -2 which are inflammatory mediators [8]. The biological activities of flavonoids have been specified to severely hinge on some elements such as the glycosylation degree, sugar residues types and following acyl esterification. Therefore, it is possible to choose different grape cultivars with a distinct flavonoid arrangement having different effects in disease protection [9]. As polyphenols are known for great antioxidant activity, their consumption may ensure some protection against neurological diseases [10]. It was noticed that people who consume three to four glasses of wine per day had an 80% decreased incidence of dementia and Alzheimer’s disease compared to those who consumed fewer or none at all [11]. Simple phenolics are also found in grapes and are mostly hydroxycinnamic acid derivatives (*p*-coumaric, caffeic, sinapic and ferulic acids) and hydroxybenzoic acid derivatives (gallic, gentisic, protocatechuic and *p*-hydroxybenzoic acids) [12]. The antibacterial activities of grape, wine and grape-derived products have been broadly considered [4,13]. It has been indicated that grape seed extract of ”Isabella” grapes var. indicate greater antibacterial activity against gram-positive bacteria in relation to the gram-negative bacteria [14]. Strong pH-dependent inhibitory effects against the growth of *Listeria monocytogenes* have been found for grape juice and skin extracts from black table grapes “Alphonse Lavallée” var., while the seed extract showed a pH-independent inhibitory effect [15]. Muscadine (*Vitis rotundifolia)* grape skin extracts have also been found to be very effective against *H. pylori* in contrast to muscadine grape seed extracts [16].

According to recent studies, defoliation has great potential to control microorganisms [17] and influence the production of biocomponents [18]. Defoliation is a powerful and regularly used action to control berry illumination and therefore flavonoid biosynthesis in berries. The obtained effect seems to depend on the defoliation timing and on the genotype [19,20,21]. Thus, defoliation can be carried out at different points in time between pre-bloom and *véraison*, but with different outcomes. Early defoliation has shown great potential for efficient control of microorganism contamination, although pre-bloom defoliation leads to reduced grain size and decreased yield of grapes [17]. Grape production and quality are reactive to heat waves, mainly at particular growth stages, such as flowering and ripening [22]. In areas with high rainfall during the winter and high temperatures during the summer season, the defoliation treatment may inhibit ripening and anthocyanin metabolism due to the high temperature of the exposed fruits. Verzera et al. [23] found that the defoliated samples from the later harvest showed the lowest amounts of anthocyanins, which could be due to excessive sunlight exposure of the berries, which causes sunburn damage and does not increase anthocyanin accumulation. Buesa et al. [24] found that post- *véraison* water stress can advance a higher concentration of phenolic substances in berry skins, and in addition, the different response to water availability of grape ripening was found in the Bobal and Tempranillo berries. Severe water stress detrimentally affected berry sugar accumulation in Tempranillo, while the opposite effect was found for Bobal. So, they concluded that the effect of vine water stress depends on the cultivar and on the severity of water stress. Vine water status was the main driver of grape ripening and these responses were genotype-dependent, while ambient temperature appeared to play a minor role in berry ripening. Lanari et al. [25] also found that late leaf removal at *véraison* negatively affected the concentration of anthocyanin and phenolic substances in Montepulciano grapevines, but not in Sangiovese. According to Zhang et al. [26], there is a strait relationship between anthocyanin biosynthesis and berry development. It starts at the *véraison* phase when the biosynthesis of anthocyanins is determined and reaches top level at berry maturity, while at the latest development stages their concentration may be noticeably decreased [27]. However, each grape species and variety has a distinct anthocyanin pattern [28]. The accumulation of tannins and hydroxycinylic acids starts already in the formation of berries and grows until the appearance of *véraison*. For grapes, the highest proportion of phenolic compounds is found in the skin, which is rich in resveratrol (3,5,4′-trihydroxy-stilbene), a polyphenol substance which shows a wide range of biological activities, such as antioxidant and antibacterial activity, and the capability to scavenge free radicals, reduce the risk of cardiovascular disease and also prevent cancer proliferation [29,30,31]. Therefore, the main goal of this study was to determine how different terms of ecologically acceptable defoliation of Blaufränkisch and Merlot grape (*Vitis vinifera* L.) leaves affects the total phenolic concentration, total and individual anthocyanin compounds, antioxidant activity and antibacterial activity on gram-positive and gram-negative human pathogens. The study was conducted during 2015 and 2016 on three treatments (control without defoliation, defoliation immediately after bloom and defoliation before the start of the *véraison* phase) in the eastern continental region of Croatia.

## 2. Results

### 2.1. Effects of Defoliation Treatments on the Concentration of Phenolic Compounds

In the case of defoliation immediately after blooming (T2), the concentration of phenolic compounds was significantly higher (U = 0; N1 = N2 = 9; *p* < 0.001) with respect to the control T1 for both grape varieties (Table 1). In the case of the Blaufränkisch variety, the concentration of phenolic compounds was significantly higher in the T2 defoliation treatment compared to the T1 and T3 treatments (U = 0; N1 = N2 = 9; *p* < 0.001). Comparison of T2 defoliation treatments of the two investigated varieties showed statistically significant higher concentrations of total phenolic concentration in the Blaufränkisch variety, compared to the Merlot variety (U = 0; N1 = N2 = 9; *p* < 0.001). However, the T1 treatments of the two investigated varieties do not differ significantly in the total phenolic concentration. In the case of defoliation before the start of the *véraison* phase (T3) the total phenolic concentration was significantly higher compared to T1 (U = 0; N1 = N2 = 9; *p* < 0.001), but less pronounced in relation to T2 (Table 1). This indicates that defoliation immediately after the blooming period is the preferable term for the Blaufränkisch variety for achieving the highest total phenolic concentration. On the other hand, defoliation before the start of the *véraison* phase in the Merlot variety also showed statistically significantly higher concentrations of total phenolic concentration in relation to T1 (U = 0; N1 = N2 = 9; *p* < 0.001), but no significant differences between the two defoliation terms were observed. Likewise, the defoliation before the start of the *véraison* phase showed statistically significant higher total phenolic concentrations in the Blaufränkisch variety, compared to the Merlot variety (U = 0; N1 = N2 = 9; *p* < 0.001). On the other hand, in the Merlot variety there are significant differences between the two different research years. A generally lower total of phenol concentrations can be noticed during the season 2016.

### 2.2. Effects of Defoliation Treatments on the Concentration of Total Anthocyanins

The total concentration of anthocyanins (Table 1), in the case of the Blaufränkisch variety showed that defoliation immediately after blooming in 2015 did not differ significantly in relation to T1. Whereas, in 2016 the T2 treatment resulted in statistically significant lower concentrations of total anthocyanins compared to T1 (U = 0; N1 = N2 = 9; *p* < 0.001). On the other hand, in the Merlot variety defoliation immediately after blooming showed statistically significantly higher concentrations of total anthocyanins compared to T1 and T3 (U = 0; N1 = N2 = 9; *p* < 0.001). The total concentration of anthocyanins was significantly increased in the case of the Blaufränkisch variety for defoliation before the start of the *véraison* phase compared to the T1 and T2 treatments (U U = 0; N1 = N2 = 9; *p* < 0.001), indicating that the preferable defoliation term for the Blaufränkisch variety for achieving the highest total anthocyanins concentration is defoliation before the start of the *véraison* phase. On the other hand, in the Merlot variety there are no significant differences between T3 and T1, indicating that the preferable defoliation term for the Merlot variety for achieving the highest total anthocyanins concentration is defoliation immediately after blooming.

### 2.3. Effects of Defoliation Treatments on the Antioxidant Activity

The differences between the two defoliation times did not prove to be significant in the Blaufränkisch variety for antioxidant activity, but the T3 in the Merlot variety resulted in significantly lower antioxidant activity compared to the T2 (U = 0; N1 = N2 = 9; *p* = 0.006) (Table 1). Thus, both defoliation terms showed statistically significant higher antioxidant activity compared to the T1 (U = 0; N1 = N2 = 9; *p* < 0.001). Defoliation before the start of the *véraison* phase showed a statistically significant higher antioxidant activity of the Blaufränkisch variety, compared to the Merlot variety (U = 0; N1 = N2 = 9; *p* < 0.001). Statistically significant differences were also observed in comparison of the groups without defoliation of the two examined varieties. The extracts of the Blaufränkisch variety showed significantly higher antioxidant activity compared to the Merlot varieties T1 extracts (U = 0; N1 = N2 = 9; *p* < 0.001).

It was found that the extracts obtained after defoliation treatments, irrespective of the defoliation time, had significantly higher antioxidant activity than without defoliation. The differences between the two defoliation times did not prove to be significant in the Blaufränkisch variety, but T2 resulted in significantly higher antioxidant activity compared to the T1 and T3 in the Merlot variety (U = 0; N1 = N2 = 9; *p* = 0.006). It could be concluded that the defoliation immediately after blooming is the preferable defoliation term for gaining better antioxidant activity of the Merlot variety extracts. Comparison of the two cultivars investigated did not show statistically significant differences in the antioxidant activity of the Blaufränkisch variety extracts relative to the Merlot variety in the case of defoliation immediately after blooming.

### 2.4. Effects Of Defoliation Treatments on Individual Anthocyanins

Quantitative analysis of individual anthocyanins based on high-performance liquid chromatography in the Blaufränkisch variety extracts in 2016 is presented in Table 2. Blaufränkisch extracts from 2015 were not analysed. Among the identified anthocyanins, eight are significantly decreased by defoliation immediately after blooming in 2016: delphinidin-3-glucoside, petunidin-3-glucoside, malvidin-3-glucoside, peonidin-3-(acetyl)glucoside, malvidin-3-(acetyl)glucoside, cyanidin-3-*O*-(6″-*p*-(coumaroyl)-glucoside, peonidin-3-*O*-(6-*p*-(coumaroyl)-glucoside and malvidin-3-*O*-(6-*p*-(coumaroyl)-glucoside (trans isomer). Defoliation before the start of the *véraison* phase did not prove to be significant in the Blaufränkisch variety extracts in 2016 (Table 2).

Defoliation immediately after blooming in the case of Merlot variety did not result in significantly different concentrations in 2015, but in 2016 resulted in significantly increased concentrations of individual anthocyanins (Table 3). Every identified anthocyanin significantly increased in concentration in 2016: delphinidin-3-glucoside, cyanidin-3-glucoside, petunidin-3-glucoside, peonidin-3-glucoside, malvidin-3-glucoside, delphinidin-3-(acetyl)-glucoside, cyanidin-3-(acetyl)-glucoside, petunidin-3-(acetyl)-glucoside, delphinidin-3-*O*-(6-*p*-(coumaroyl)-glucoside, peonidin-3-(acetyl) glucoside, malvidin-3-(acetyl) glucoside, cyanidin-3-*O*-(6″-*p*-(coumaroyl)-glucoside, petunidin-3-*O*-(6-*p*-(coumaroyl)-glucoside, malvidin-3-*O*-(6-*p*-(coumaroyl)-glucoside (cis isomer), peonidin-3-*O*-(6-*p*-(coumaroyl)-glucoside and malvidin-3-*O*-(6-*p*-(coumaroyl)-glucoside (trans isomer).

Defoliation before the start of the *véraison* phase in the case of the Merlot variety resulted in significantly decreased concentrations of individual antocyanins in 2015 compared to T2 and T1, but in 2016 did not prove to be significant (Table 3), as with the Blaufränkisch variety extracts in 2016. Among the identified anthocyanins, nine of them significantly decreased concentration in 2015: delphinidin-3-glucoside, petunidin-3-glucoside, malvidin-3-glucoside, delphinidin-3-*O*-(6-*p*-(coumaroyl)-glucoside, peonidin-3-(acetyl) glucoside, malvidin-3-(acetyl) glucoside, petunidin-3-*O*-(6*-p*-(coumaroyl)-glucoside, malvidin-3-*O*-(6-*p*-(coumaroyl)-glucoside (*cis* isomer) and malvidin-3-*O*-(6-*p*-(coumaroyl)-glucoside (*trans* isomer).

### 2.5. Effects of Defoliation Treatments on Antibacterial Activity

The best effect in gaining stronger antibacterial activity of Blaufränkisch and Merlot grape skin extracts was obtained by defoliation immediately after blooming in the Blaufränkisch variety, as it resulted in a significant decrease of MIC values in all four investigated bacteria (Table 4). In general, the MIC values where higher in 2016, but it can be seen that the MIC values were almost two times higher in 2016 for *P. aeruginosa* and *S. aureus* in the Blaufränkisch variety, and for *S. aureus* in the Merlot variety. In the case of the Blaufränkisch variety, defoliation immediately after blooming showed statistically significantly stronger antibacterial activity compared to the defoliation before the start of *véraison*, and also compared to the group without defoliation. On the other hand, in the Merlot variety defoliation immediately after blooming showed statistically significant stronger antibacterial activity compared to the group without defoliation (U = 0; N1 = N2 = 9; *p* < 0.001), but not compared to the defoliation before the start of *véraison*, except for *P. aeruginosa* and *S. aureus*. Defoliation before the start of *véraison* resulted in a significant decrease of MIC values in all four investigated bacteria. On the other hand, in the Merlot variety defoliation before the start of *véraison* did not prove to be significant.

## 3. Discussion

**Figure 1:**
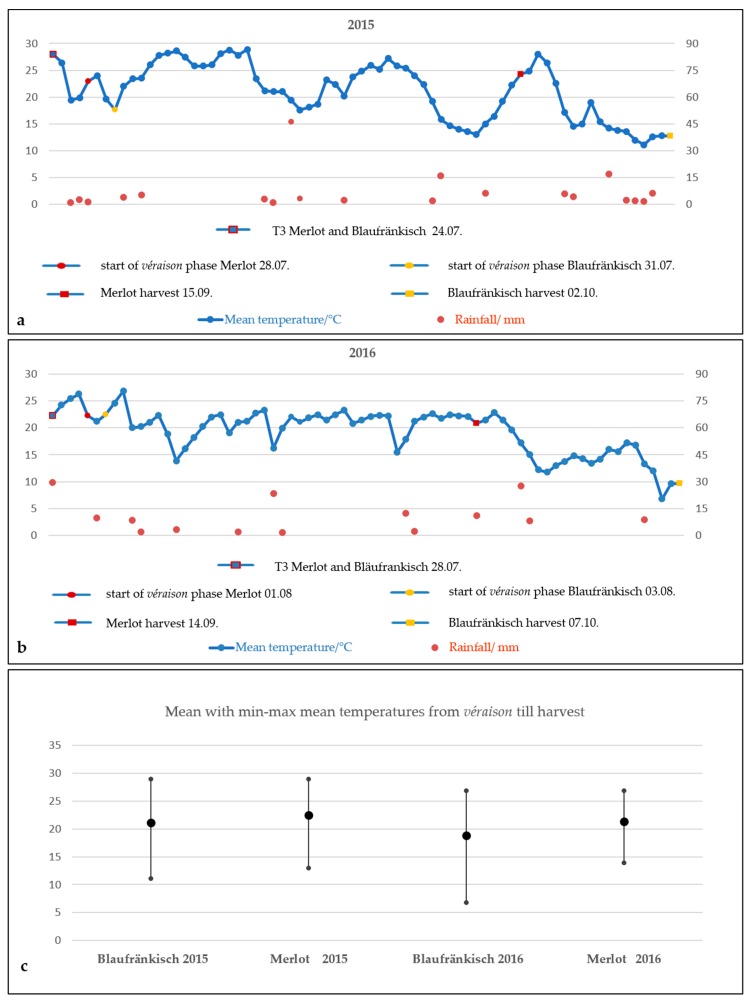
(**a**) Mean temperature (°C, left axis) and rainfall (mm, right axis) trend from T3 treatment till harvest date in 2015; (**b**) Mean temperature and rainfall trend from T3 treatment till harvest in 2016; (**c**) Mean with min-max mean temperatures from *véraison* till harvest date.

Our previous work [32] showed that early leaf removal treatments resulted in statistically significant increased total phenolic concentration, total anthocyanin compounds, and antioxidant activity measured in extracts of grape skin. Therefore we performed ecologically acceptable defoliation of Blaufränkisch and Merlot grape (*Vitis vinifera* L.) leaves to investigate the effect of two different times of defoliation: immediately after bloom and before the start of the *véraison* phase. The timing of partial defoliation is proven to affect the grape vegetative response. The research of Hunter et al. [22] confirmed that the earlier the defoliation was applied, the more the lateral shoot length and the number of lateral shoots increased, resulting in a higher total shoot length. While if defoliation was performed at *véraison* it had no effect on lateral growth. In our research, defoliation immediately after blooming increased the total phenolic concentration for both grape varieties, achieved the highest total anthocyanins concentration and antioxidative activity in the case of the Merlot variety, and achieved the best effect in gaining stronger antibacterial activity of the Blaufränkisch and Merlot grape skin extracts. The relationship between berry skin composition and defoliation seems to be cultivar dependent. The increased total phenolic concentration after the defoliation treatment performed prior to blooming was noted by Poni et al. [33]. According to research carried out by Risco et al. [34] early defoliation of the *Vitis vinifera* L., Nero d’Avola variety resulted in an increase of the total content of anthocyanin, flavonoids and polyphenols [23]. Thus, results also showed that overall vine performance was not stimulated by the defoliation treatments. Mijowska et al. [35] found that early defoliation, particularly pre-flowering, resulted in a significant increase in total polyphenol content. Results from the research of Jerman et al. [36] on grape varieties of Pinot Noir indicate that early defoliation increases antioxidant activity. The research carried out by Radovanović et al. (2015) showed that the early defoliation of the Cabernet Sauvignon variety resulted in a significant increase in total phenol concentrations of up to 88.75%, as well as an increase in antioxidant activity up to 12.70% compared to the control group. The total concentration of anthocyanins in the case of the Blaufränkisch variety showed different trends between two different research years. Defoliation immediately after blooming in 2015 did not differ significantly in relation to the control group, but in 2016 the treatment resulted in statistically significant lower concentrations of total anthocyanins. Also, a generally lower total phenol concentration can be noticed during 2016 in the Merlot variety. This is in accordance with previous research, that the weather conditions have a considerable impact on the amounts of antioxidant components in grape skin extracts [32]. As it can be seen from the Table 5 and Figure 1 because the cumulative rainfall was much higher, and min-max temperatures were lower for 2016, it could be concluded that precipitation is an important climatic factor in terms of the formation of phenolic compounds in the skin of grapes [37]. The increase in the number of days with warm temperatures is particularly relevant for vineyards also. According to Ferrer et al. [38] and Barbagallo et al. [39], the concentration of individual anthocyanins has shown the utmost alteration between the years. Our results of decreased concentration of anthocyanins with excess rainfall during the vegetation period in 2016 are in accordance with results of Ferrer et al. [38] and Roby et al. [40] where water deficits increased the amount of skin anthocyanins. Berry temperature is important, as it is affected not only by sunlight exposure but also by the availability of water to maintain transpiration. Sunlight exposure, combined with simultaneous high temperatures, leads to a decrease in phenolic compounds [6]. Jerman et al. [36] followed an air temperature/precipitation behaviour in addition to a grape surface temperature monitoring during an experimental trial of early leaf removal of Pinot Noir’ grapes from Vipava Valley. The temperatures of the grapes were consistently higher (4 °C on average) in comparison to atmospheric ones, but apparently not affected the synthesis of phenols, since the increases of total anthocyanins and polyphenols till the mid of August were observed. Interestingly, their concentrations have decreased afterwards, most likely due to a precipitation-related grape phenols dilution effect. All these environmental factors can have an effect on the development of vines and their particular phenological stages, with consequences on berry quality, but the precise response is dependent on the grapevine genotype [19]. Therefore, it appears that the response to leaf removal may vary depending on the cultivar. In our research, defoliation before the start of *véraison* resulted in a significant increase in total anthocyanins concentration and antioxidative activity in the case of the Blaufränkisch variety, in contrast to the Merlot variety. This also resulted in a significant decrease of MIC values in all four investigated bacteria, but less pronounced compared to the defoliation immediately after blooming. However, the best antibacterial activity in general (Table 4) was found with the Merlot variety after defoliation immediately after blooming in 2015, despite the lower determined values of TAC and TPC compared to Blaufränkisch. This may be due to a greater number of individual anthocyanin components in Merlot or complex mixtures of plant metabolites, such as alkaloids, saponins, tannins, amino acids, triterpenoids, phlobatannins, steroids and catechol found in grape skin [41]. Secondary metabolite distribution may also be modified during plant growth, or linked to the changes of climatic conditions, so in future studies it would be of great interest to perform phytochemical screening. The defoliation performed by Intrigliolo et al. [42], at the full maturity of grapes on the Mandó cultivar from Southeast Spain resulted in a reduction in grain size, but also increased concentrations of phenol, anthocyanin and tannins in grape berries. In red grape varieties anthocyanin accumulation starts from *véraison* and achieves its limits in the finite phases of fruit maturation when the synthesis runs down [43], but phenolic synthesis and accumulation in grape berry is also determined by genetic factors and the interaction between genotype and environment [44,45]. It can be concluded that the defoliation treatment results in an increased phenolic concentration in the grape skin, but whether different terms of leaf removal will result in different increases in phenolic concentration is highly specific for each individual grape variety. Given that no previous research has been carried out thus far to describe the effect of defoliation treatment on the antibacterial activity of grape skin extracts, this work is unique. Previous studies have investigated the antibacterial activity of grape skin extracts but without considering the effect of defoliation treatment, especially in different terms. Nirmala and Narendhirakannan [41] found that *Vitis vinifera* L. Muscat from the Coimbatore district of Tamil Nadu, India are rich in antioxidants and possess antimicrobial activity. Katalinić et al. [46] conducted a study in which they studied the antibacterial effect of grape skin extracts of 14 *V. vinifera* varieties grown in Dalmatia on gram-positive and gram-negative bacteria. Lower MIC values were confirmed for phenolic mixtures from white grape cultivars for all tested species which was contrary to expectations. The investigated grape skin extracts showed good antibacterial as well as antioxidant activity, which are of paramount importance when it comes to human health. The positive effects of grapes in the form of prevention of the occurrence of certain diseases, or at least their progression, are very interesting and relevant for research. It would be desirable to compare different extraction methods with solvents of different polarity or supercritical fluid extraction, combined with the effect of various new food technologies such as ultrasound, high hydrostatic pressure and pulsating electric field treatments.

## 4. Materials and Methods

### 4.1. Plant Material and Experimental Design

Two *V. vinifera* varieties, Blaufränkisch (VIVC-Variety-No.: 1459) and Merlot Noir (VIVC-Variety-No.: 7657), were studied during 2015 and 2016. A vineyard, planted in 2013, is situated at the transition from the luvisol to the stagnic luvisol, located in Mandićevac (lat. 45. 368 417, lon. 18. 245 611, elevation 208 m), in the eastern continental region of Croatia, the subregion of Slavonija, among the vineyards of Đakovo, with south exposure and a general fall W→E of 9.8%. The chemical properties of this soil indicate an acid reaction. The vine training system was single Guyot, with 12 buds per plant. The vines were planted with 2.2 m spacing between rows and 0.8 m within rows, for a total of 5681 vines/ha. The experiment was designed by random block formation consisting of three replicates. The study was conducted on three treatments: control (T1), and two different treatments of six basal leaf removal were performed: defoliation immediately after bloom: BBCH 69 phase (T2) and defoliation before the start of *véraison* was performed during BBCH 79 phase (T3) according to the extended BBCH scale [47]. The treatments included ten plants per every separate treatment in three repetitions, which was 90 vines in total. The experimental field did not supply an irrigation system. Rainfall and mean temperatures for 2015 and 2016 during the March–October time period were obtained from the Meteorological and Hydrological Service of Croatia and presented in Table 5. Cumulative rainfall during the April–September period in 2016 was higher than in 2015, while the mean temperatures were equivalent. The trend of mean temperatures from the T3 treatment till harvest date can be seen in the Figure 1a,b, for 2015 and 2016, respectively, while mean min-max temperatures for both years from the *véraison* phase till harvest date can be seen in the Figure 1c.

### 4.2. Grape Skin Extraction

Grape skin extraction was performed according to the method described by Rustioni et al. [48]. Each sample represents more randomly selected clusters from which ten berries were separated. The grape skin was manually detached from the pulp and samples were gently dried with a napkin to avoid loss of anthocyanins. Thereafter, the mixture was soaked for 20 h with 20 mL of a mixture of ethanol: water: hydrochloric acid (70:29:1, *w/w/w*). Each sample represents more randomly selected clusters from which 10 berries were separated in each repetition. The total phenolic concentration from the extract was determined after filtration. The extraction procedure was carried out with three biological replicates.

### 4.3. Determination of Total Phenolic Concentration (TPC)

The total phenolic concentration of grape skin extracts was determined by a spectrophotometric method which used the Folin–Ciocalteu reagent. The standard calibration (0.018–0.50 mg mL^−1^) curve was plotted using gallic acid [49]. The results were derived from triplicate analyses, normalized against negative control of solvent and expressed as milligrams of gallic acid equivalents (GAE) per gram of grape skin and reported as mean ± standard deviation (SD).

### 4.4. Determination of Total Anthocyanins (TAC)

The total anthocyanins were determined by a spectrophotometric method described by Nagel and Wulf [50]. Absorbance of each dilution was measured at the 540 nm, against a blank with distilled water. The total anthocyanin concentration results were derived from triplicate analyses, normalized against negative control of solvent and expressed in mg of malvidin-3-*O*-glucoside equivalents (MAE) per g of grape skin, using the molar extinction coefficient (ε) of malvidin-3-*O*-glucoside of 28,000 L mol^−1^ cm^−1^ and molar weight (MW) (493.2 g mol^−1^) and reported as mean ± standard deviation (SD).

### 4.5. 2,2-Diphenyl-1-Picrylhydrazyl (DPPH) Radical Scavenging Activity

The total antioxidant activities of grape skin extracts were determined using the DPPH radical scavenging assay described earlier [51]; 750 μL of the diluted grape skin extracts (average final concentration 100 mg mL^−1^ for Merlot variety and 150 mg mL^−1^ for Blaufränkisch variety) was mixed with the same amount of 0.2 mM DPPH radical solution, so the final DPPH radical concentration was 0.1 mM. The mixture was well stirred and incubated at room temperature for 30 min. Ascorbic acid (AA) was used as a reference compound in the concentration range 2–200 μg mL^−1^. All experiments were performed in triplicate. The absorbance decrease at 517 nm was measured, and DPPH scavenging activity was established using Equation (1):

DPPH activity = (A_b_ + A_s_) − A_m_)/A_b_ × 100 (1)

where A_b_ is the absorbance of 0.1 mM DPPH radical solution at λ = 517 nm, A_s_ is the absorbance of 0.1 mM extraction solution at λ = 517 nm, and A_m_ is the absorbance of 0.1 mM solution mixture of tested extracts and DPPH radical at 517 nm.

### 4.6. High-Performance Liquid Chromatography (HPLC): Separation and Detection of Anthocyanins

The anthocyanins were analysed according to OIV-MA-AS315-11 with some modifications [52]. Anthocyanins were analysed by direct injection of the samples, previously centrifuged and filtered through a 0.45 μm pore size membrane filter, in an Agilent 1100/1200 series HPLC system equipped with an Agilent photodiode array detector (Agilent Technologies, Wilmington, NC, USA). The separation was performed on a reversed phase column: LiChrospher 100 RP 18 (5 µm) in LiChroCart 250–4 with guard column LiChroCart 4 mm RP 18 all obtained from Merck (Darmstadt, Germany). Separation was performed with solvents: (A) water/formic acid/acetonitrile (87:10:3, *v/v/v*) and (B) water/formic acid/acetonitrile (40:10:50, *v/v/v*). The following gradient of eluents was used: 6%–30% (B) 0–15 min, 30%–50% (B) 15–30 min, 50%–60% (B) 30–35 min, 60%–6% (b) 35–41 min. The flow rate was 0.4 mL min^−1^, detection wavelength 520 nm and the analysis were performed at 20 °C. Individual anthocyanins were identified by comparing their retention times with external standards and literature [53,54,55,56]. Diglycosides were detected in very low and not relevant amounts and they were excluded accordingly. Available standard substances of cyanidin-3,5- diglucoside, cyanidin-3-glucoside, delphinidin-3-glucoside, malvidin-3,5-diglucoside, petunidin- 3-glucoside, peonidin-3-glucoside, malvidin-3-glucoside, all obtained from Sigma-Aldrich (St. Louis, MO, USA). Anthocyanins were quantified by using a seven-point external calibration curve (R^2^ = 0.9997) obtained by injecting standard solutions of malvidin-3-monoglucoside chloride. Results were expressed as malvidine-3-glucoside equivalents MAE per gram of grape skin. All analyses were done in triplicate and results expressed as mean ± standard deviation (SD).

### 4.7. Antibacterial Susceptibility Testing

#### 4.7.1. Microorganisms and Growth Conditions

*Bacillus subtilis* and *Staphylococcus aureus* as two gram-positive, and *Escherichia coli* and *Pseudomonas aeruginosa* as gram-negative bacterial strains, were used to determine the antibacterial activities of the grape skin extracts. These bacteria were isolates from various clinical specimens obtained from the Microbiology Service of the Public Health Institute of Osijek–Baranja County (Osijek, Croatia). Working cultures were prepared from subcultures and grown overnight in Muller Hinton Broth (MHB) (Fluka Analytical, Buchs, Switzerland) under optimal conditions (37 °C with 50% humidity). The antibacterial standard Amikacin sulfate (Biorisan, Amikacin, Vocate, Glyfada, Greece) was dissolved in sterile distilled water.

#### 4.7.2. Minimum Inhibitory Concentrations

MIC values were determined by a modified microdilution method [57] as described in our previous work [58]. Briefly, a total of 100 μL of midlogarithmic-phase bacterial cultures (5 × 10^5^ CFU mL^−1^) in Mueller Hinton Broth were added to 100 μL of two-fold serially diluted extracts (250–0.122 mg mL^−1^). Wells containing bacterial inoculum without extracts (growth control) and wells containing only broth and solvent (background control) were included in each plate. The antibacterial standard amikacin sulfate was co-assayed under the same conditions. The MIC value was defined as the lowest concentrations of extract at which there was no color change or visual turbidity due to microbial growth, derived from triplicate analyses, normalized against negative control and expressed as micrograms per milliliter.

### 4.8. Statistical Data Processing

All statistical analysis were achieved by the statistical program STATISTICA 12.0 (Statsoft, Inc., Tulsa, OK, USA). The normality of the distribution of numeric variables was tested by the Shapiro–Wilk test. Given that the data does not follow normal distribution, the nonparametric Mann–Whitney U-test was used. All tests were performed at a level of significance of α = 0.05.

## 5. Conclusions

Both defoliation treatments significantly increased the total phenolic concentration as well as the antioxidant and antibacterial activity of the grape skin extracts in both examined *Vitis vinifera* L. varieties Blaufränkisch and Merlot. Defoliation just after blooming is the preferable defoliation term in the Merlot variety for achieving the highest total anthocyanin concentration, gaining the highest antioxidant activity and a significant increase of antibacterial activity against all four investigated bacteria. Defoliation before the start of the *véraison* phase is the preferable defoliation term for achieving the highest total anthocyanin concentration in the Blaufränkisch variety. In general, the treatment of defoliation immediately after bloom was more successful under the given experimental conditions compared with the defoliation before the start of the *véraison* phase.

## Figures and Tables

**Table 1:** Total phenolic concentration (TPC) in grape skin extracts expressed as mg of GAE per g of grape skin, Total anthocyanins concentration (TAC) in grape skin extracts expressed as mg of MAE per g of grape skin and 2,2-diphenyl-1-picrylhydrazyl (DPPH) radical scavenging activity expressed as % DPPH radical scavenging activity of Blaufränkisch and Merlot variety.

*Vitis vinifera* L. variety	Year	Treatment	TPC (mgGAE g_skin_^−1^)	TAC (mgMAE g_skin_^−1^)	DPPH Radical Scavenging Activity (%)
Blaufränkisch	2015	T1	12.79 ± 0.59_a_A^c^	2.06 ± 0.16_a_A^b^	54.03 ± 0.53_b_A^b^
		T2	18.2 ± 0.06_a_A^a^	1.93 ± 0.11_a_A^b^	56.52 ± 0.91_b_A^a^
		T3	17.26 ± 0.53_a_A^b^	2.37 ± 0.09_a_A^a^	55.86 ± 0.31_b_A^a^
	2016	T1	12.86 ± 0.26_a_A^c^	1.98 ± 0.09_b_A^b^	56.34 ± 0.84_a_A^b^
		T2	17.12 ± 1.02_a_A^a^	1.72 ± 0.03_b_A^c^	57.69 ± 0.85_a_A^a^
		T3	15.61 ± 0.18_a_A^b^	2.35 ± 0.11_a_A^a^	57.06 ± 0.08_a_A^a^
Merlot	2015	T1	12.85 ± 0.22_a_A^b^	1.60 ± 0.06_a_B^b^	51.97 ± 0.34_b_B^c^
		T2	14.97 ± 0.31_a_B^a^	1.84 ± 0.04_a_A^a^	57.18 ± 0.49_a_A^a^
		T3	14.96 ± 0.63_a_B^a^	1.62 ± 0.05_a_B^b^	54.04 ± 0.97_b_B^b^
	2016	T1	7.73 ± 0.33_b_B^b^	1.02 ± 0.07_b_A^b^	52.86 ± 1.55_a_B^c^
		T2	9.29 ± 0.27_b_B^a^	1.32 ± 0.04_b_A^a^	57.50 ± 0.63_a_A^a^
		T3	8.36 ± 0.13_b_B^a^	1.09 ± 0.12_b_A^b^	56.59 ± 0.30_a_B^b^

Different green subscript lower case letters in each row indicate statistically significant differences (*p*< 0.001) between years for the same variety and same treatment. Different blue upper case letters in each row indicate statistically significant differences (*p* < 0.001) between two varieties for the same year and same treatment. Different magenta superscript lower case letters in each row indicate statistically significant differences (*p* < 0.001) between treatments for the same variety and same year.

**Table 2:** Individual anthocyanins concentration in grape skin extracts expressed as mg of MAE per g of grape skin for Blaufränkisch variety.

Skin anthocyanins (mg_MAE_ g _skin_^−1^)	Blaufränkisch					
Year	2015			2016		
Treatment	T1	T2	T3	T1	T2	T3
Delphinidin-3-glucoside	n.d.	n.d.	n.d.	0.0665 ± 0.0102 ^a^	0.0498 ± 0.0058 ^b^	0.059 ± 0.0095 ^a,b^
Cyanidin-3-glucoside	n.d.	n.d.	n.d.	0.0312 ± 0.0107 ^a^	0.0337 ± 0.0095 ^a^	0.0403 ± 0.0101 ^a^
Petunidin-3-glucoside	n.d.	n.d.	n.d.	0.0867 ± 0.0069 ^a^	0.0605 ± 0.0048 ^b^	0.0762 ± 0.0071 ^c^
Peonidin-3-glucoside	n.d.	n.d.	n.d.	0.3372 ± 0.0585 ^a^	0.3098 ± 0.0656 ^a^	0.3961 ± 0.0587 ^a^
Malvidin-3-glucoside	n.d.	n.d.	n.d.	0.9323 ± 0.0547 ^a^	0.5611 ± 0.0459 ^b^	0.8167 ± 0.1464 ^a^
Petunidin-3-(acetyl)-glucoside	n.d.	n.d.	n.d.	0.0005 ± 0.0004 ^b^	0.0005 ± 0.000 ^b^	0.0009 ± 0.0004 ^a^
Delphinidin-3-*O*-(6-*p*-(coumaroyl)-glucoside	n.d.	n.d.	n.d.	0.0028 ± 0.0013 ^b^	0.0022 ± 0.0001 ^b^	0.0031 ± 0.0005 ^a^
Peonidin-3-(acetyl)-glucoside	n.d.	n.d.	n.d.	0.0021 ± 0.0002 ^a^	0.0016 ± 0.0003 ^b^	0.0023 ± 0.0005 ^a^
Malvidin-3-(acetyl)-glucoside	n.d.	n.d.	n.d.	0.0120 ± 0.0013 ^a^	0.0059 ± 0.0012 ^b^	0.0110 ± 0.0036 ^a^
Cyanidin-3-*O*-(6″-*p*-(coumaroyl)-glucoside	n.d.	n.d.	n.d.	0.0035 ± 0.0003 ^a^	0.0025 ± 0.0004 ^b^	0.0039 ± 0.0006 ^a^
Petunidin-3-*O*-(6*-p*-(coumaroyl)-glucoside	n.d.	n.d.	n.d.	0.0041 ± 0.0023 ^b^	0.0034 ± 0.0001 ^b^	0.0047 ± 0.0006 ^a^
Malvidin-3-*O*-(6-*p*-(coumaroyl)-glucoside (*cis* isomer)	n.d.	n.d.	n.d.	0.0029 ± 0.0018 ^b^	0.0016 ± 0.0001 ^b^	0.0027 ± 0.0008 ^a^
Peonidin-3-*O*-(6-*p*-(coumaroyl)-glucoside	n.d.	n.d.	n.d.	0.0252 ± 0.0005 ^a^	0.0165 ± 0.001 ^b^	0.0221 ± 0.0039 ^a^
Malvidin-3-*O*-(6*-p*-(coumaroyl)-glucoside (*trans* isomer)	n.d.	n.d.	n.d.	0.073 ± 0.0108 ^a^	0.0377 ± 0.0104 ^b^	0.0638 ± 0.0229 ^a^
∑ c Anthocyanins (with unknown)	n.d.	n.d.	n.d.	1.5908 ± 0.0237 ^a^	1.0944 ± 0.0357 ^b^	1.5144 ± 0.109 ^a^

Different superscript lower case letters in each row indicate statistically significant differences (*p* < 0.001) between treatments.

**Table 3:** Individual anthocyanins concentration in grape skin extracts expressed as mg of MAE per g of grape skin for Merlot variety.

Skin anthocyanins (mg_MAE_ g _skin_^−1^)	Merlot					
Year	2015			2016		
Treatment	T1	T2	T3	T1	T2	T3
Delphinidin-3-glucoside	0.1384 ± 0.0186 ^a^	0.1382 ± 0.0022 ^a^	0.1247 ± 0.0004 ^b^	0.0424 ± 0.0023 ^b^	0.0633 ± 0.0014 ^a^	0.0397 ± 0.0114 ^b^
Cyanidin-3-glucoside	0.0207 ± 0.0032 ^a^	0.0202 ± 0.0048 ^a^	0.0201 ± 0.0043 ^a^	0.0115 ± 0.0014 ^b^	0.0184 ± 0.0035 ^a^	0.0140 ± 0.0026 ^b^
Petunidin-3-glucoside	0.1347 ± 0.0134 ^a^	0.1353 ± 0.0032 ^a^	0.1168 ± 0.0067 ^b^	0.043 ± 0.0016 ^b^	0.0674 ± 0.0093 ^a^	0.0422 ± 0.008 ^b^
Peonidin-3-glucoside	0.0687 ± 0.0138 ^a^	0.072 ± 0.0143 ^a^	0.0611 ± 0.0031 ^a^	0.0457 ± 0.008 ^b^	0.0753 ± 0.0087 ^a^	0.0534 ± 0.0066 ^b^
Malvidin-3-glucoside	0.5212 ± 0.0436 ^a^	0.5293 ± 0.0143 ^a^	0.4169 ± 0.0507 ^b^	0.2048 ± 0.0113 ^b^	0.3318 ± 0.0482 ^a^	0.1948 ± 0.0197 ^b^
Delphinidin-3-(acetyl)-glucoside	0.0201 ± 0.0029 ^a^	0.021 ± 0.0023 ^a^	0.0186 ± 0.0026 ^a^	0.0051 ± 0.0013 ^b^	0.0079 ± 0.0014 ^a^	0.0048 ± 0.0013 ^b^
Cyanidin-3-(acetyl)-glucoside	0.003 ± 0.0004 ^a^	0.0036 ± 0.0009 ^a^	0.0032 ± 0.001 ^a^	0.0008 ± 0.0001 ^b^	0.0015 ± 0.0004 ^a^	0.0009 ± 0.0005 ^b^
Petunidin-3-(acetyl)-glucoside	0.0207 ± 0.0026 ^a^	0.0207 ± 0.0018^a,b^	0.0178 ± 0.003 ^a^	0.0051 ± 0.0019 ^b^	0.0086 ± 0.0008 ^a^	0.0052 ± 0.0008 ^b^
Delphinidin-3-*O*-(6-*p*-(coumaroyl)-glucoside	0.0305 ± 0.0024 ^a^	0.0301 ± 0.0015 ^a^	0.0251 ± 0.004 ^b^	0.0067 ± 0.0002 ^b^	0.0104 ± 0.001 ^a^	0.0062 ± 0.0008 ^b^
Peonidin-3-(acetyl)-glucoside	0.0085 ± 0.0022 ^a^	0.0084 ± 0.0031 ^a^	0.0048 ± 0.0031 ^b^	0.0047 ± 0.0006 ^b^	0.0065 ± 0.0025 ^a^	0.0041 ± 0.0016 ^b^
Malvidin-3-(acetyl)-glucoside	0.1077 ± 0.0123 ^a^	0.093 ± 0.0322 ^a^	0.0513 ± 0.033 ^b^	0.0338 ± 0.0028 ^b^	0.0558 ± 0.0072 ^a^	0.0298 ± 0.0035 ^b^
Cyanidin-3-*O*-(6″-*p*-(coumaroyl)-glucoside	0.0095 ± 0.0013 ^b^	0.015 ± 0.0053 ^a^	0.0127 ± 0.0073 ^a^	0.0052 ± 0.0008 ^b^	0.0081 ± 0.0009 ^a^	0.0052 ± 0.0006 ^b^
Petunidin-3-*O*-(6*-p*-(coumaroyl)-glucoside	0.0298 ± 0.0022 ^a^	0.029 ± 0.0012 ^a^	0.0184 ± 0.0089 ^b^	0.0068 ± 0.0005 ^b^	0.0107 ± 0.0012 ^a^	0.006 ± 0.0006 ^b^
Malvidin-3-*O*-(6-p-(coumaroyl)-glucoside (*cis* isomer)	0.0037 ± 0.0004 ^a^	0.0041 ± 0.0002 ^a^	0.0022 ± 0.0013 ^b^	0.0016 ± 0.0003 ^b^	0.0025 ± 0.0003 ^a^	0.0013 ± 0.0004 ^b^
Peonidin-3-*O*-(6-p-(coumaroyl)-glucoside	0.0203 ± 0.0019 ^a^	0.0226 ± 0.0032 ^a^	0.0479 ± 0.061 ^a^	0.0116 ± 0.0015 ^b^	0.0181 ± 0.0004 ^a^	0.0098 ± 0.0011 ^c^
Malvidin-3-*O*-(6*-p*-(coumaroyl)-glucoside (*trans* isomer)	0.1753 ± 0.0157 ^a^	0.166 ± 0.0069 ^a^	0.097 ± 0.057 ^b^	0.0455 ± 0.0013 ^b^	0.0746 ± 0.0047 ^a^	0.0365 ± 0.0062 ^c^
∑ c Anthocyanins (with unknown)	1.3222 ± 0.1247 ^a^	1.318 ± 0.0299 ^a^	1.0471 ± 0.0597 ^b^	0.4783 ± 0.0241 ^b^	0.7667 ± 0.0806 ^a^	0.4572 ± 0.0475 ^b^

Different superscript lower case letters in each row indicate statistically significant differences (*p* < 0.001) between treatments.

**Table 4:** Minimum inhibitory concentrations (MIC) of grape skin extracts against *Escherichia coli*, *Pseudomonas aeruginosa, Bacillus subtilis*, and *Staphylococcus aureus* (mg mL^−1^).

*Vitis vinifera* L. variety	Year	Treatment	MIC (mg mL^−1^)			
			*E. coli*	*P. aeruginosa*	*B. subtilis*	*S. aureus*
Blaufränkisch	2015	T1	12.79 ± 0.52_a_A^a^	6.23 ± 0.49_b_A^a^	12.46 ± 0.99_a_A^a^	12.46 ± 0.79_b_A^a^
		T2	9.15 ± 0.16_a_A^c^	4.71 ± 0.24_b_A^c^	9.37 ± 0.45_a_A^c^	9.61 ± 0.79_b_A^c^
		T3	9.96 ± 0.34_a_A^b^	5.05 ± 0.11_b_A^b^	10.06 ± 0.24_a_A^b^	9.96 ± 0.34_b_A^b^
	2016	T1	12.79 ± 0.53_a_A^a^	12.79 ± 0.54 _a_A^a^	12.46 ± 0.10_a_A^a^	25.93 ± 0.65_a_A^a^
		T2	9.15 ± 0.17_a_A^c^	9.15 ± 0.18_a_A^c^	9.37 ± 0.46_a_A^c^	18.22 ± 0.29_a_A^c^
		T3	9.96 ± 0.35_a_A^b^	9.96 ± 0.36_a_A^b^	10.06 ± 0.25_a_A^b^	19.93 ± 0.69_a_A^b^
Merlot	2015	T1	7.72 ± 0.66_a_B^a^	4.03 ± 0.36_a_B^a^	7.73 ± 0.67_a_B^a^	7.61 ± 0.58_b_B^a^
		T2	6.88 ± 0.68_a_B^b^	3.27 ± 0.39_a_B^b^	6.84 ± 0.51_a_B^b^	7.14 ± 0.12_b_B^b^
		T3	7.31 ± 0.63_a_B^a,b^	3.82 ± 0.49_a_B^a^	7.31 ± 0.63_a_B^a,b^	7.28 ± 0.25_b_B^a,b^
	2016	T1	7.74 ± 0.67_a_B^a^	4.13 ± 0.37_a_B^a^	7.83± 0.68_a_B^a^	15.78 ± 1.29_a_B^a^
		T2	6.98 ± 0.69_a_B^a^	3.47 ± 0.40_a_B^b^	6.94 ± 0.52_a_B^a^	13.75 ± 1.26_a_B^b^
		T3	7.41 ± 0.64_a_B^a,b^	3.92 ± 0.50_a_B^a^	7.41 ± 0.64_a_B^a,b^	15.18 ± 0.37_a_B^a^
Amikacine sulfate			0.000016	0.00003	0.000016	0.000008

Different green subscript lower case letters in each row indicate statistically significant differences (*p* < 0.001) between years for the same variety and same treatment. Different blue upper case letters in each row indicate statistically significant differences (*p* < 0.001) between varieties for the same year and same treatment. Different magenta superscript lower case letters in each row indicate statistically significant differences (*p* < 0.001) between treatments for the same variety and same year.

**Table 5:** Rainfall and mean temperatures in Đakovo from March to October in 2015 and 2016.

Month	Mean Monthly Temperature, °C		Mean Min-Max Temperature, °C		Rainfall, mm	
	2015	2016	2015	2016	2015	2016
March	7.8	7.9	2.7–15.0	3.3–17.6	48.5	82.7
April	12.7	13.7	5.9–19.7	5.8–19.2	18.2	46.5
May	18.2	16.6	12.0–25.4	9.4–23.8	130.8	72.1
June	21.3	21.4	13.5–27.0	17.1–28.4	16.8	92.7
July	25.0	23.3	17.6–30.0	14.0–28.4	12.4	125.2
August	24.2	21.0	17.6–28.9	16.9–26.8	64.9	51.0
September	18.2	18.4	11.1–28.0	11.8–22.8	63.1	61.5
October	11.3	10.4	11.1–17.7	5.6–17.2	114.6	66.0
Mean temp., (°C)	17.33	16.58				
Cumulative rainfall, mm					469.3	597.7
